# Factors Determining the Success and Failure of eHealth Interventions: Systematic Review of the Literature

**DOI:** 10.2196/10235

**Published:** 2018-05-01

**Authors:** Conceição Granja, Wouter Janssen, Monika Alise Johansen

**Affiliations:** ^1^ Future Journal Norwegian Centre for E-health Research Tromsø Norway; ^2^ Telemedicine and E-health Research Group University of Tromsø-The Artic University of Norway Tromsø Norway

**Keywords:** telemedicine, eHealth, medical informatics, systematic review, success, failure

## Abstract

**Background:**

eHealth has an enormous potential to improve healthcare cost, effectiveness, and quality of care. However, there seems to be a gap between the foreseen benefits of research and clinical reality.

**Objective:**

Our objective was to systematically review the factors influencing the outcome of eHealth interventions in terms of success and failure.

**Methods:**

We searched the PubMed database for original peer-reviewed studies on implemented eHealth tools that reported on the factors for the success or failure, or both, of the intervention. We conducted the systematic review by following the patient, intervention, comparison, and outcome framework, with 2 of the authors independently reviewing the abstract and full text of the articles. We collected data using standardized forms that reflected the categorization model used in the qualitative analysis of the outcomes reported in the included articles.

**Results:**

Among the 903 identified articles, a total of 221 studies complied with the inclusion criteria. The studies were heterogeneous by country, type of eHealth intervention, method of implementation, and reporting perspectives. The article frequency analysis did not show a significant discrepancy between the number of reports on failure (392/844, 46.5%) and on success (452/844, 53.6%). The qualitative analysis identified 27 categories that represented the factors for success or failure of eHealth interventions. A quantitative analysis of the results revealed the category quality of healthcare (n=55) as the most mentioned as contributing to the success of eHealth interventions, and the category costs (n=42) as the most mentioned as contributing to failure. For the category with the highest unique article frequency, workflow (n=51), we conducted a full-text review. The analysis of the 23 articles that met the inclusion criteria identified 6 barriers related to workflow: workload (n=12), role definition (n=7), undermining of face-to-face communication (n=6), workflow disruption (n=6), alignment with clinical processes (n=2), and staff turnover (n=1).

**Conclusions:**

The reviewed literature suggested that, to increase the likelihood of success of eHealth interventions, future research must ensure a positive impact in the quality of care, with particular attention given to improved diagnosis, clinical management, and patient-centered care. There is a critical need to perform in-depth studies of the workflow(s) that the intervention will support and to perceive the clinical processes involved.

## Introduction

In the last decades, it has been a challenge for policy makers to ensure access to healthcare to populations living in rural and remote areas [1]. Additionally, global demographic trends, such as the increasing number of elderly people, have been changing healthcare delivery due to a growing demand for long-term care and increasing costs [2-5]. Against this background, eHealth has been presented as a solution [6-8]. In the context of this study, we define eHealth as the use of information and communication technology in healthcare.

A vast amount of eHealth interventions have been reported to fail during clinical implementation [9,10]. Tanriverdi and Iacono [8] demonstrated that a considerable amount of research with promising results did not contribute to clinical practice. Berg [11] stated more specifically that 75% of implemented eHealth should be considered a failure.

According to Bashshur et al [12], the assessment of eHealth interventions rests on three pillars of care: (1) access, (2) quality, and (3) cost containment. They describe these three pillars as the promises that eHealth interventions are required to fulfill to attain a successful outcome and, indeed, that each of these promises must be met. Considering the aforementioned reports on the failure of eHealth interventions [8-11], it appears reasonable to assume that the promises represented by these three pillars are not often accomplished.

To improve the success of eHealth, it is important to identify the factors that can influence, positively or negatively, the outcome of the intervention. Such factors can vary from project-specific to recurring issues, with the three pillars proposed by Bashshur et al [12] expected to have an important role in the success or failure of eHealth interventions. However, as the field of medical informatics is positioned between the fast-changing field of informatics and the rather conservative field of healthcare, organizational and operational aspects can be expected to play an important part in the outcome of eHealth interventions.

The overall aim of this study was to seek, through a systematic review, patterns in the assessment of eHealth intervention outcomes, and through these patterns to identify factors that can help explain why eHealth interventions fail or succeed in clinical practice. Therefore, we systematically searched for original studies that provide data to address the following key questions:

Key question 1: According to reports in abstracts, why are eHealth interventions failing to achieve the expected results and foreseen benefits? Specifically, (1) What are the major facilitators and barriers contributing to the implementation of eHealth? (2) How are these facilitators and barriers contributing to the adoption of eHealth? (3) Are the perceived facilitators and barriers to eHealth adoption similar among the study participants?

Key question 2: According to the literature, what is the most relevant factor regarding the possible outcome in terms of success or failure, or both? Specifically, in what manner is this factor affecting the adoption of eHealth?

## Methods

This systematic review was guided by the *Cochrane Handbook* [13], and the reporting is based on the Preferred Reporting Items for Systematic reviews and Meta-Analyses (PRISMA) guidelines [14]. We established the review methods before conducting the review, and the reports did not justify any deviations from the protocol.

### Search Strategy

We searched the PubMed database in October 2016 for original articles published in English up to this date. The main reason for using only the PubMed search engine was the availability of a vast amount of articles in eHealth research from both a medical and a sociological standpoint. In this way, we expected that we would find most of the relevant clinical outcomes for this study. Since the review focused on implemented eHealth tools, we did not consider the inclusion of articles from a technology perspective to be necessary. As this strategy proved to be useful, we searched no other academic databases.

We performed an initial search using the Medical Subject Heading (MeSH) telemedicine AND challenges, based on prior knowledge obtained from articles that referenced eHealth success or failure, which identified 658 articles. We evaluated the title and abstract of these articles and identified possible search terms. We determined the term “lessons” to be important and used it together with the previous search, identifying 63,299 articles. We analyzed the resulting articles to further specify the search terms and define the search string following the patient, intervention, comparison, and outcome (PICO) framework [15].

We based the search string on three classes of the PICO framework, since the class comparison did not apply to this study. We defined these classes as (P): healthcare; (I): eHealth; and (O): change, failure, or success. We combined all terms in each class with the logical operator OR and linked the classes using the logical operator AND. Over the course of refining the search results, we tried 2 search strings. In the first, we used the search terms extracted from the previous search to create a PICO scheme, constrained to the last 10 years, which identified 11,950 articles. In the second, we removed the time constraint and refined the search terms to focus on eHealth interventions that were used in actual clinical practice. This last search string (Textbox 1) was the one we used to retrieve the articles used in the review process.

**The search string developed according to the patient, intervention, comparisona, and outcome (PICO) framework. Comparison (C) was not applicable to this study.**

**Healthcare (P)**

((clinical practice[Title/Abstract] OR real use[Title/Abstract] OR real practice[Title/Abstract] OR clinical implication OR health care effect[Title/Abstract] OR health care impact[Title/Abstract] OR practical trials[Title/Abstract] OR clinical trials[Title/Abstract] OR practical clinical implementation[Title/Abstract] OR practical clinical trials[Title/Abstract] OR implemented service[Title/Abstract] OR adoption[Title/Abstract] OR adoption rate[Title/Abstract]))

**eHealth (I)**

((((“telemedicine”[MeSH] OR medical informatics[TIAB] OR eHealth[Title/Abstract] OR ehealth[Title/Abstract] OR telemedicine[Title/Abstract] OR telehealth[Title/Abstract] OR mhealth[Title/Abstract] OR mhealth[Title/Abstract] OR health telematics[Title/Abstract] OR tele-health[Title/Abstract] OR etherapy[Title/Abstract] OR wireless health[Title/Abstract] OR healthcare technology[Title/Abstract] OR telecare[Title/Abstract] OR medical information system[Title/Abstract] OR telemonitoring[Title/Abstract] OR telepresence[Title/Abstract] OR electronic health information[Title/Abstract] OR teleconsultation[Title/Abstract] OR teleintervention[Title/Abstract] OR e-rehabilitation[Title/Abstract])))

**Change, failure, or success (O)**

((fail*[Title/Abstract] OR succes*[Title/Abstract] OR barrier*[Title/Abstract] OR interoperability[Title/Abstract] OR usability[TIAB] OR lessons learned[Title/Abstract] OR implications[Title/Abstract] OR experiences[Title/Abstract] OR implementation[Title/Abstract])))

**Reading interpretation guidelines for the abstract review.**

- Funding from government or tax money equals influence on society.
- Policies have an effect on both organizations and availability of tools for patients.
- Coordination and interoperability problems have consequences for patients, professionals, and systems.
- Extra (or changes in) work is seen as workflow.
- Safety is a relative term, interpreted as compared with traditional ways.
- Workforce problems are interpreted as change of workflow.
- (Un)familiarity with tools is seen as information technology training.
- Paternalism and empowerment is seen as empowerment or engagement.
- Medical (studies) students are seen as health professionals.
- Time is seen as either workflow or costs, depending on the context.

We included no articles based on hand searches of reference lists for the reasons outlined under Section 10.2.2.3 of the *Cochrane Handbook* [13]: “positive studies are more likely to be cited” and “retrieving literature by scanning reference lists may thus produce a biased sample of studies.”

### Study Selection

We analyzed the titles and abstracts of the articles that resulted from the final search string for inclusion according to the following predefined exclusion criteria: not an original work; unclear or no results; not research; not in English. We defined the exclusion criteria based on the key questions to include original studies in healthcare-related fields, with a focus on success and failure, that reported on a form of eHealth or medical informatics, but which did not have to be the main goal or result but should have been a key component. Textbox 2 lists the reading interpretation guidelines for the abstract review.

### Data Collection and Synthesis

A qualitative analysis [16,17] was carried out by 2 of the authors (CG and WJ) to classify the outcomes reported in the articles’ abstract according to the following 3 levels.

#### Category

The category level was evidence that the factor described in the abstract contributed to the success or failure of the eHealth intervention. We defined categories based on the information found in the abstracts (presented in the Results section below). We chose this strategy to minimize the risk of bias, since predetermined categories could have led to a model that merely reflected our opinion.

#### Success and Failure

This level indicated whether the identified category was described as a success or failure factor reported in the intervention outcome narrative. As success and failure are important concepts of this study, we explain our considerations on the terms here.

We classified the factors in the categories as *success* if they were considered to facilitate the achievement of the study goals. The same category may have been described as success and failure in the same study by different participants. If specific features of an intervention were mentioned to be a success, we attributed these to success, even when the overall project was classified as failure.

**Further exclusion criteria of articles.**

- The category is mentioned only in the text.
- No extensive analysis of the category or reporting on the effects on eHealth outcomes is present.
- The category is only identified as relevant to the success or failure of eHealth, and no further considerations are taken.
- The article is not in English.

We classified the factors in the categories as *failure* if they were considered to be barriers to achieving the study goals. Different participants may have described the same category as success and failure in the same study. If specific features of an intervention were mentioned to be a failure factor, we attributed these to failure, even when the overall project was classified as success.

#### Entity

This level referred to the role of the study participant who reported the identified factor in the categories. The entities could assume the following values.

In this review, *patients* were people who received care. Therefore, we also included clients (ie, people with less-urgent problems) and customers (ie, people who were interested in monitoring their own health). This entity also included people who gave care to patients in a nonprofessional context (ie, parents, family, and friends).

*Healthcare professionals* comprised all people who provided care services in a professional context. This included physicians, nurses, therapists, mental health workers, and other professional groups trained in providing care. It did not necessarily have to be direct care, but they had to have been providing care to patients.

The *health system* included management and supporting staff, infrastructure, the technological health systems (both software and hardware), and ideological systems such as national health plans and systems.

The *society* value included participants who were described as potential users and were not identified as belonging to the entities described above.

*All* involved all the above entities, and we classified the category identified in the reports as success and failure.

### Full-Text Analysis

In addition to categorizing abstracts for success and failure factors of eHealth interventions, we analyzed the full text for the category with the highest frequency of unique articles according to the categorization results. Such analysis was aimed at gathering the data that provide knowledge related to key question 2.

Articles in which the category with the highest unique article frequency was reported to contribute to the success or failure of the eHealth intervention were eligible for a full-text review. However, in the course of the full-text assessment, we refined the selection according to the exclusion criteria presented in Textbox 3.

In summary, abstract and full-text reviews were conducted independently by 2 authors (CG and WJ), who extracted data based on the inclusion and exclusion criteria into a structured a Microsoft Excel spreadsheet. The 2 authors analyzed all abstracts a second time to confirm the categorization, identified all relevant factors reported to contribute to the success or failure of eHealth interventions, and noted them in the categorization model. In this manner, every time a new factor was identified, a new category was created. The full-text review was conducted with special attention to the descriptions of how the category with the highest unique article frequency was affecting the success of the eHealth intervention. All disagreements were resolved by consensus discussions.

## Results

The search string identified 903 articles, 7 of which we excluded, as they were duplicates or incomplete. The titles and abstracts of the remaining 896 articles were read by 2 of the authors (CG and WJ) for compliance with the exclusion criteria described in the Methods section, and 221 were included in the study.

**Figure 1:**
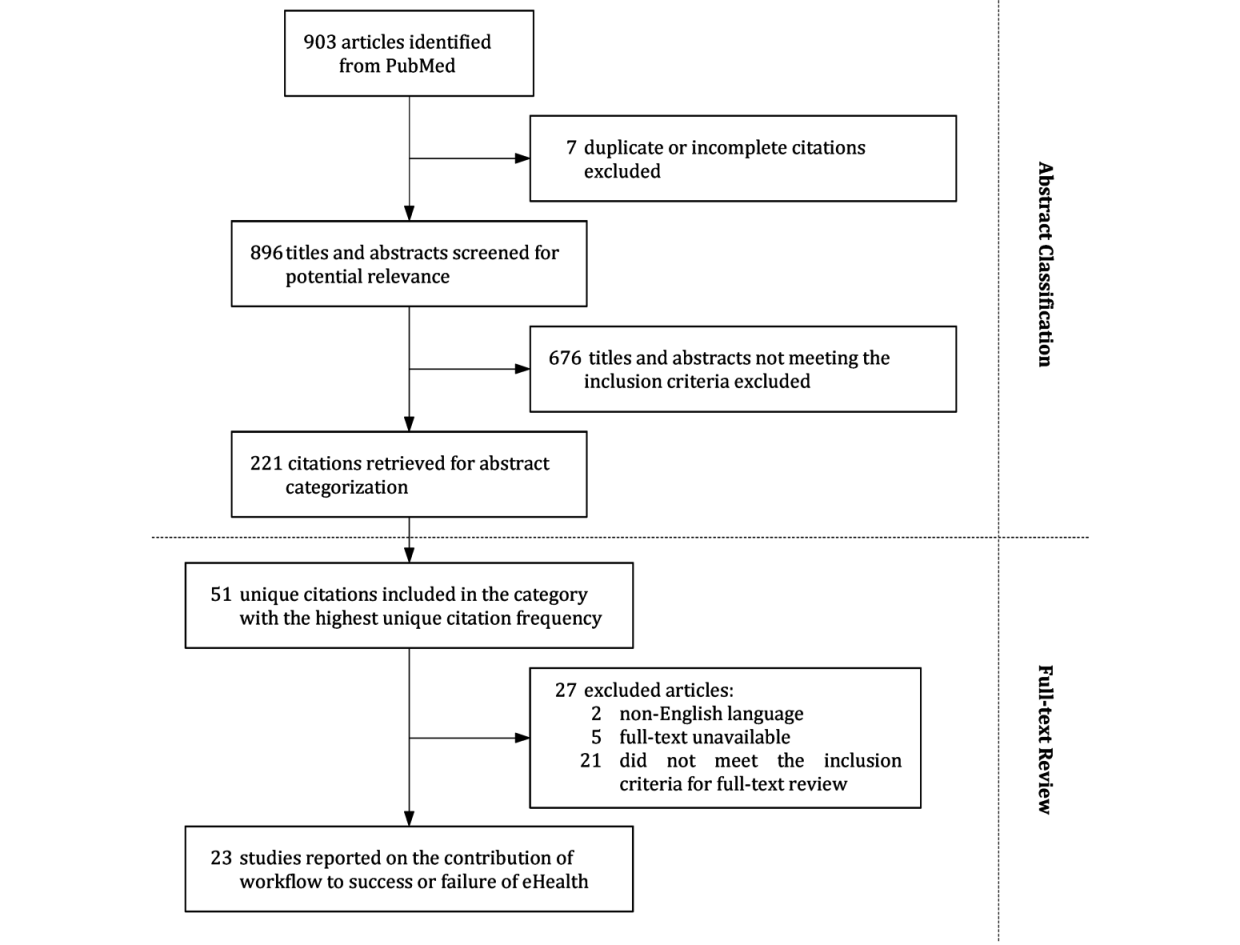
Preferred Reporting Items for Systematic reviews and Meta-Analyses (PRISMA) flowchart of the literature search and article selection.

Figure 1 presents the literature search and selection process based on the PRISMA guidelines [14].

### Key Question 1 (Barriers and Facilitators)

We defined the categories included in the categorization model based on the information we found in the abstracts. We identified 27 categories, which are summarized and defined in Table 1 [18].

We classified the abstracts of the 221 articles according to the 3 levels described in the Methods section. Multimedia Appendix 1 [19-239] presents the outcomes.

Furthermore, we analyzed the article frequency, as Multimedia Appendix 2 shows.

The narratives on the category (quality of healthcare) most mentioned as contributing to the success of eHealth interventions reported on improved diagnosis [28,72], better communication with the patient [48,84], and supported patient-centered care [19,48]. Factors less clinically related were also mentioned, such as the diminishment of the care provision gap for patients [22], and the improvement of patients’ clinical management [25].

On the other hand, a few articles in the category (costs) most mentioned as contributing to the failure of eHealth interventions established a relationship between the costs and quality of care. As an example, Chan et al [46] pointed out as the main benefit of the eHealth intervention focus of the study that patients in a rural area could be examined by physicians from central hospitals without needing a physical consultation, while at the same time resulting in financial savings. However, the main focus of the articles in this category was on eHealth adoption. Villalba et al [102] identified national investments and funding programs as facilitators in the adoption of 11 eHealth interventions in 8 European countries. O’Toole et al [140], Devriendt et al [45], and Foldy [108] also identified the shortage of financial resources as a common barrier to the adoption and implementation of eHealth. In a similar manner, Ford et al [114] stressed the importance of finding financial mechanisms to support the organizational changes required to adopt eHealth. While acknowledging that a national policy for investment in eHealth interventions is crucial to its adoption, Rozenblum et al [132] argued that financial incentives should be based on patient outcomes that might ensue from the eHealth intervention. DeWorsop et al [129] and Lee and Billings [133] reported on the importance of cost effectiveness to promoting the adoption of eHealth interventions.

To better understand what were, for each entity, the most relevant categories reported to contribute to the success and failure of eHealth interventions, Table 2 summarizes the results from the article frequency analysis. We excluded the society entity from this analysis, as we did not consider the number of articles to be representative.

### Key Question 2 (Success/Failure Factors)

The article frequency analysis, presented in Multimedia Appendix 2, revealed that the most representative category was workflow, with 51 unique articles. We assessed all the unique articles according to the exclusion criteria presented in Textbox 2, which resulted in 23 articles remaining for full-text review (Figure 1).

The full-text analysis of the 23 articles, with special attention to the descriptions of how the workflow category affected the success of the eHealth intervention, identified the following 6 barriers.

*Workload* increased the amount of work and tasks (or time required to perform them) needed to complete a clinical process, when compared with the workflow established before the eHealth intervention [19,22,36,69,73,85,87,93,95,98,99,107].

*Workflow disruption* resulted in the inability to complete the work process in a linear and smooth manner [19,69,73,84,92,106].

**Table 1:** Categories and their definitions.

Category	Definition
Usability	Ease of use, learnability (ie, easy for users to learn how to perform basic tasks), and interface intuitiveness are present.
Adoption	Users acknowledge the use of the eHealth tool.
Workflow	Workflow is defined by the way people interact with their work, communication pathways, and other people. It should be noted that different professional groups might have different understanding of workflow. As most of the selected abstracts were based on sociological research, this definition excludes the logistics of information flows.
Costs	This category includes all articles that reported on money, finances, and value in financial contexts.
System architecture	This refers to the fundamental organization of a system embodied in its components, their relationships to each other and to the environment, and the principles guiding its design and evolution [18].
Policies	The policies category is essentially related to governmental policies and rules. It often involves legal and financial aspects based on subsidies to promote the use of eHealth tools.
Interoperability	This refers to the ability of a system to exchange and make use of information from another system.
Patient empowerment and self-management	These are tools or techniques that give patients control over their own health and access to their health data.
Infrastructure	In the context of this study, infrastructure refers to the communication structures required for the operation of the eHealth tool.
Leadership	This refers to all managerial levels and the decisions made by them.
Assessment	This category covers considerations of feasibility, efficiency, effectiveness, operational results or other associated outcomes, and the effects of the implementation of eHealth tools.
Conformity with other healthcare entities	Conformity refers to the usability of information between healthcare providers in regard to clinical processes and the ability to replicate the eHealth tool implementation in different sites.
ICT^a^ training	This covers user-focused training and support in the use of the eHealth tool.
Holistic approach	When the focus is on patient care, this approach implies perceiving the subject of study as a whole person, considering mental and social factors, as opposed to just someone who contracted a disease or disability. When the holistic approach focuses on the organization, this is seen as whole rather than separate entities (ie, departments, wards, and different forms of special care).
Reliability of connection and technology	This refers to the stability of communications structures during use. The stability and reliability of eHealth tools, both software and hardware related, are also included.
Standardization	Software and hardware conform to standards.
Culture	This comprises the culture of an organization, country, region, or population group.
ICT vs traditional methods	This category considers the comparability between the use of eHealth tools (ICT), and preestablished methods (traditional) (eg, videoconferencing vs face-to-face consultations).
Privacy and security	Privacy refers to the confidentiality of personal information, usually relating to personal data stored on computer systems. Security refers to the protection of computer systems against information, communications, and physical damage. In the course of classifying the abstracts, reports on security issues were often related to privacy problems in healthcare settings. Therefore, these 2 categories were combined.
Legal	Legal problems relate to legislation issues.
Safety	Safety is considered from a clinical perspective.
Access to healthcare	Access refers to the right of or opportunity for patients to receive, or come in contact with, healthcare organizations or providers.
Education	Education enlightens people about their health (eg, providing information on their disease or disability).
Quality of healthcare	A good quality of healthcare improves the healthcare delivery process and its outcomes, in both an organizational and a clinical context.
Patient-provider relationship	healthcare professionals and patients.
User involvement	This is considered from the human-centered design perspective.
Adherence to treatment	This refers to the patient’s compliance with the treatment plan.

^a^ICT: information and communication technology.

**Table 2:** Categories contributing to the success or failure of eHealth interventions by entity.

Outcome	Entity		
	Patients	Healthcare professionals	Health system
Success	Patient empowerment and self-management	Quality of healthcare	Costs Policies
Failure	Privacy and security	Workflow	Costs

*Alignment with clinical processes* was a barrier when the eHealth tool did not integrate with or support the existing clinical process [38,85].

*Undefined and changed roles* resulted when the responsibility for a workflow task was not the same after the eHealth intervention, or new tasks were included in the workflow and no responsibilities were assigned [22,36,87,95,97,105,106].

*Undermined face-to-face communication* refers to the impact on personal contact with the patient and other healthcare professionals [56,72,86,87,91,106].

*Staff turnover* refers to the rotation of healthcare professionals between departments, or short-term contracts, that require new learning or training on the eHealth tool [103].

Workload can be classified as the biggest workflow-related concern, since it was overrepresented in the results, being addressed in 12 of the 23 studies. In these studies, healthcare personnel stressed the increase in the amount of work after the implementation of the eHealth tool. eHealth was described as being both time and resource intensive [19,22,36,69,73,85,98,99,107] and [19,87,93,95] indicated discontent about the amount of self-reported and self-recorded health data provided by the tool for assessment.

The second most mentioned barrier, addressed in 7 studies, was the undefined roles and change of work practice of the parties involved in the workflow. Narratives reported, for instance, that the new role was tangential to their role as healthcare professionals [22,36,87]—for example, coaching patients in the use of the technology, analyzing the self-reported data and subsequently answering the patient’s questions [87,95], the need for new competences [97], and unresolved attribution of responsibilities [105,106].

Workflow disruption was significantly present in the studies, reflected in narratives describing eHealth as not being fitted to the existing workflow due to time (eg, data provided to the system a priori, and work tasks having to be performed by others) [19,73,84,106] or space [92] constraints, and breaking of traditions [69].

According to Kapadia et al [83], healthcare personnel report a preference for face-to-face communication over digital long-distance systems. Nielsen and Mathiassen [38] mentioned the loss of contact between personnel as a trigger to the reduction of knowledge sharing and collegial relationships. Less mentioned, but still significant, were statements that eHealth is impersonal and, therefore, undermines face-to-face communication [56,72,86,87,91,106], substantiated by the claim that the foundation of good nursing is physical presence, human touch, and the use of all senses.

Less addressed in the studies, but still significant barriers, were the alignment of the eHealth intervention with the clinical workflow [38,85] and staff turnover [103]. These are related to how supportive and well integrated the tool is in the workflow and the need for constant training of staff, respectively.

In the literature, two general workflows were mentioned. The first was the preestablished workflow, defined as the workflow in an organization, or at a specific organizational level (eg, the cardiology ward in a hospital), before the eHealth intervention. The second was the new workflow, which describes the workflow after the eHealth intervention. Different authors have various ideas about this change.

Some [38,84,85,92] argued that eHealth interventions should be adapted into the preestablished workflow in order to succeed. Others [19,72,73 93,97] advocated that the workflow will change, or is necessary to change, in order for the intervention to be successful.

eHealth interventions in healthcare organizations triggered changes in the workflow [73,97]. Such changes were not limited to the directly involved staff, but also had an impact on others within the organization [73]. Therefore, changes in the workflow had the potential to alter the organization both in a negative and in a positive way [36,38,86,88,103]. Additionally, a change that was initially a positive development could lead to a rather negative outcome. Such circumstances are reported by Das et al [86], Davis et al [87], and Chung et al [95], where the overabundance of data led to an inability to use the data due to time constraints.

Professional values and personal feelings should also be considered to be barriers to eHealth interventions, as they may come into conflict with the use of technology [36,38]. In these works, feelings of healthcare staff about the technology resulted in negative thoughts and skepticism, resulting in the technology never fully being integrated into the workflow.

## Discussion

### General Findings

There is a reasonable amount of original research exploring the effects of eHealth interventions. However, among the 903 articles we identified, only 221 met the inclusion criteria for this systematic review. The studies were heterogeneous by country, type of eHealth intervention, method of implementation, and reporting perspectives (ie, patient, healthcare professionals, health system, and society). The article frequency analysis presented in Multimedia Appendix 2 did not find a significant discrepancy between the number of reports on failure (392/844, 46.5%) and success (452/844, 53.6%), which encourages definitive conclusions on the key questions that prompted the review.

While evaluating the 903 articles, we realized that the studies could be grouped into three chronological eras, according to their research focus: (1) up to 1999: in this first era, most of the articles addressed the technology with a focus on aspects such as functionality and infrastructure; (2) 2000-2009: in the second era, the focus shifted from the technology to the organization; in this setting, an organization could be a healthcare organization or a community (local, regional, national, or international); (3) 2010 to the present: the third era focused on individuals, where researchers investigated how people work, often from a bottom-up perspective, compared with a top-down scope on the complete organization.

The first two eras defined in this chronological pattern are supported by Nielsen and Mathiassen [38].

When we applied the three chronological eras to the 221 articles selected for abstract classification, it became clear that the search strategy achieved the desired results in identifying studies with relevant clinical outcomes. This is demonstrated by the fact that most of the articles (168 articles) fell into the third era, and only 3 articles were included in the first era, which had a technological focus.

### Key Question 1 (Barriers and Facilitators)

The category most mentioned as contributing to the success of eHealth interventions was quality of healthcare. This category was also one of the three pillars of care described by Bashshur et al [12] as being the support of successful eHealth interventions. The authors related quality of care to professional performance standards, related the role and contribution of the intervention to clinical practice, and described how this contribution is achieved [12]. This is in line with the narratives found in the articles assigned to this category.

Most of the unique articles in the category quality of healthcare belonged in the third era, with only 9 being in the second era, and none in the first. Belonging in the third era, the focus was on the individual, which could explain the increase in focus on quality of care.

The articles in the quality of healthcare category were evenly distributed among the entities, showing a significant difference between the article frequency as success (n=55) and failure (n=12), and revealing a positive view among the users, thus positioning this category as an evident facilitator of eHealth interventions.

On the other hand, costs is the category most mentioned as contributing to the failure of eHealth interventions. Similar to quality of healthcare, this category is also one of the three pillars of the promises of eHealth proposed by Bashshur et al [12]. In their work, the cost containments pillar focused on cost reductions for patients and providers in a broad sense, supporting a clear definition of how the eHealth intervention would facilitate the provision of care services at a lower cost without loss of quality of care [12]. However, the main focus of the articles in the costs category was on eHealth adoption, and only a few articles established a relation between the costs and quality of care in the manner of Bashshur et al.

The classification of unique articles in the costs category according to the aforementioned eras revealed that most, 80% (40 articles), fell in the third era. In line with the focus on the individual that characterized this era, we noted that only 13 of the 50 unique articles included results after the eHealth intervention, and that as many as 37 report on expectations, potentials, and other future possibilities. Most of these articles arose from the social sciences and addressed the actors’ expectations of eHealth, revealing the general idea that eHealth interventions bring cost reductions along with the implementation. An important aspect of expectations is that they are based on the actors’ opinions or wishes, often before the results are clear. However, the actual costs and financial benefits can be measured only after implementation. Since most research was done during the implementation, often at an early stage, this means that data were being collected at a stage when funding was still available and the full impact of the eHealth intervention on the organization was not yet evident. This being said, the costs category certainly presents as a barrier to eHealth interventions.

Looking at the category’s article frequency distribution among the entities, costs was mostly mentioned by the health system entity, being, in fact, considered as the most important to both the success and the failure of eHealth interventions. Such lack of a relevant difference between the contribution of costs to success (18 articles) and failure (19 articles) leads to the conclusion that this category is a major concern for the health system. Thus, and since this entity includes organizations and governmental bodies, we conclude that certain financial conditions are required to distribute eHealth services. Hence, we inferred that, to adopt eHealth in their services, it is not important for the health system to attain financial profit, but rather to not have a loss. It is not surprising to see that, along with costs, the category most cited as contributing to that success of eHealth interventions for the health system entity was policies, as the importance of national policy investments and reimbursement rules was so often mentioned in the articles.

However, such perspective on what is most relevant for the success and failure of eHealth interventions is not shared by the other entities. Considering the narratives related to quality of healthcare, described in the Results section, it is not surprising that this category appeared as the most important for the success of eHealth interventions for healthcare professionals. Following the same line of thought, workflow appeared as the most relevant for the failure of eHealth. We discuss the ways in which each of these issues affected the outcome of eHealth interventions below under Key Question 2. However, we can already assert that what these issues have in common is the deprivation of time to provide care services. Conversely, patients considered of most importance that the eHealth interventions support them in managing their own health independently. This is demonstrated in that the most cited category by the patient entity as contributing to the success of eHealth interventions was patient empowerment and self-management. It seems almost too obvious to state that, as patients want to be provided with the means to manage their own health, their most mentioned category as leading to the failure of eHealth was privacy and security. This reveals that, even though patients want to be independent and manage their own health remotely, they are aware of the sensitivity of the data that are being shared.

### Key Question 2 (Success/Failure Factors)

Kruse et al [240] examined barriers in eHealth research on a quantitative basis for several consecutive years. In their work, they stated that workflow was one of the most mentioned barriers in literature, which is confirmed by the article frequency analysis presented in Multimedia Appendix 2, where workflow is the category with the most unique articles. This positions this category as being of major concern for the entities.

The claim that eHealth interventions should be fitted to the preestablished workflow in order to succeed [38,84,85,92] was also upheld by Gardner [241] and in the systematic review by Kawamoto et al [242]. In opposition, others [19,72,73,93,97] supported that changes in the workflow are inevitable and necessary for the eHealth intervention to be successful, stressing that the adoption of new eHealth tools within the preestablished workflow creates problems during the implementation process [19]. This is corroborated by the findings in the systematic review by Davis et al [243] and by Bowens et al [244], who also point out that the need to reengineer the workflow to integrate eHealth can be a trigger to improve efficiency, distribution of tasks, patient safety, and the quality of the data collected from the patient. Such disparity of opinions leads to 2 questions. (1) What is the importance of eHealth to workflow? (2) How are the design and the outcomes of eHealth related?

#### What is the Importance of eHealth to Workflow?

Healthcare organizations’ resistance to change has identified barriers to eHealth interventions [245,246]. Most organizations were not created to accommodate eHealth and, as noted by Appelbaum and Wohl [247], the inability of healthcare organizations to adapt to changes is a barrier to their own sustainability. The resistance to change is also present at the staff level. Pardo del Val and Martinez Fuentes [248] proposed a framework for the sources to resistance to change, among which are the “relation between change values and organizational values,” denoting the difference between what is important for the individual and for the organization, and “cynicism,” denoting the negative feelings toward the success of the change [249]. This is in line with the narratives on how professional values and personal feelings hinder the use of eHealth [36,38], found in the articles selected for review.

To maximize the likelihood of a successful eHealth intervention, healthcare professionals must acquaint themselves with the tool [250]. Furthermore, there should not be a possibility to fall back on the old workflow [39,250].

#### How Are the Design and the Outcomes of eHealth Related?

If eHealth does not meet the expectations or requirements of healthcare personnel, it is possible that the eHealth tool will not be used as anticipated [38]. This is caused by a difference between the prospects, which are often made by management personnel, and the reality of healthcare personnel. In the literature, the technical perspective during the design phase is identified as a significant contributor to the gap between prospect and reality [94,251]. Declerck and Aime [251] used the term technocentrism to classify the importance of technology in the design phase, where other actors, such as healthcare professionals, appear as secondary concerns. In addition, it should be taken into consideration that different professions within healthcare have different needs from eHealth [83], and these needs have to be addressed to make eHealth applications operate successfully in healthcare organizations.

In a veiled manner of recognizing that the technical focus during the design phase was a barrier to eHealth adoption, the technology design mentality shifted to one with a holistic and human focus. There is a widespread perception in human-computer interaction that recognizes that users should be involved in the design in order to create technology that is more relevant, resulting in the wide acceptance of user-centered design and participatory design approaches [252]. Thus, it is legitimate to conclude that the design and the outcomes of eHealth are clearly related, and that user involvement during the design phase is of the most importance for the success eHealth interventions within the domain of workflow.

### Conclusions to Key Question 1

With this systematic review, we identified quality of healthcare as the major facilitator of eHealth interventions and costs as the major barrier.

Within the quality of healthcare category, a positive impact in clinical care appears to be relevant to the adoption of eHealth. The impact is assessed with varied metrics, such as improved diagnosis, clinical management, and patient-centered care. The role of the costs category in the adoption of eHealth seems to be more consensual. Most of the studies included in this category identify the shortage of financial resources as a common barrier to the adoption and implementation of eHealth.

The importance of quality of healthcare in the success of eHealth interventions is shared only by the healthcare professionals entity. The concern of healthcare professionals with care services is also reflected by the workflow category being the most mentioned contributor to the failure of eHealth interventions.

The patient entity revealed their wish of controlling their own health by placing the patient empowerment and self-management character of the intervention as the major contributor to success. However, this wish for control does not come without apprehension, as demonstrated by mentioning the privacy and security category as the major contributor to the failure of eHealth.

The health system entity attributed to 1 category, costs, the same relevance to both success and failure of eHealth interventions. This does not appear as a surprise, as this category mostly relates to financial resources. As these financial resources often originate from governmental sources, it is reasonable that the health system entity mentioned the policies category, along with costs, as a major contributor to the success of eHealth interventions.

### Conclusions to Key Question 2

According to the reviewed literature, workflow was the most relevant factor to the outcome of eHealth interventions across all entities. A full-text review identified 6 barriers to the adoption of eHealth related to this category. Most of these barriers were reported by the healthcare professionals entity, and are as follows: (1) workload, (2) workflow disruption, (3) alignment with clinical processes, (4) undefined and changed roles, (5) undermined face-to-face communication, and (6) staff turnover.

### Limitations

Some limitations in this review must be acknowledged. Despite having searched the PubMed database using standard systematic review protocols, we could have searched further databases. This might have limited the results, as the search process may not have captured relevant studies that were not indexed in the PubMed database. However, we considered that the PubMed search alone yielded a representative overview of the field.

Additionally, many studies were based on data that were gathered either before or during the implementation process when the eHealth intervention was not well established in the workplace. As the reports were collected at such an early stage, they might be overshadowed by emotional resistance and fear of change.

### Future Research

In spite of the limitations, this systematic review remains a useful source of information, as it synthesized common challenges in the development of eHealth interventions and in the planning of their implementation. In this regard, considerations for future research include the following:

- Identify potential facilitators and barriers at the earliest possible stage to ensure that full account is taken when defining the development and implementation strategy;
- Determine the impact of eHealth interventions on the quality of care, with particular attention given to improved diagnosis, clinical management, and patient-centered care;
- Evaluate the financial needs and consequences in the short, medium, and long term, to avoid nonadoption due to lack of funding;
- Perform in-depth studies of the workflow(s) that the intervention will support and perceive the clinical processes involved;
- Improve the privacy and security features of eHealth interventions targeted at patients.

## Appendix

Multimedia Appendix 1 Article abstract classification.

Multimedia Appendix 2 Article frequencies.

## Abbreviations

MeSH: Medical Subject Heading
PICO: patient, intervention, comparison, and outcome
PRISMA: Preferred Reporting Items for Systematic reviews and Meta-Analyses
