# Supplementary material for: Factors Determining the Success and Failure of eHealth Interventions: Systematic Review of the Literature
Source: J Med Internet Res. 2018 May 1;20(5):e10235. doi: 10.2196/10235 (PMC5954232; doi:10.2196/10235)
Supplement: Multimedia Appendix 1 [file jmir_v20i5e10235_app1.pdf]

Classification of the resulting articles' abstract according to three levels: Category, entity [Patient (P), Health care professionals (HP), Health system (HS), Society (Soc) and All] and contribution to the e-health intervention success (S) and/or failure (F).

	**Entity**								
**Category**	**P**		**HP**		**HS**		**Soc**		**All**
	**S**	**F**	**S**	**F**	**S**	**F**	**S**	**F**	
**Access to healthcare**	[24, 25, 34, 43, 111, 143, 218, 222, 226]	[22, 72]	[25, 34, 52, 56, 64, 91, 166, 188, 226]	[22, 40, 56, 88, 208]	[188, 234]	[22, 62, 193]		[22]	[141, 201]
**Actor engagement**	[67, 78, 100, 165, 184]	[147, 194]	[38, 67, 85, 100, 114, 196, 237, 238]	[85, 132, 147, 194, 237]	[38, 67, 100, 140, 196, 239]	[62, 194, 239]	[100]		[66, 71, 92, 109, 189]
**Adherence / loyalty to treatment**	[112, 129, 157]	[187]	[143]						[107]
**Adoption**	[22, 26, 47, 48]	[37, 48-50]	[24, 40, 44, 47, 51-54]	[50-52, 55-58]	[40, 59]	[50, 55, 60-63]	[64]		[65-71]
**Assessment**	[22]		[22]	[136]	[22]	[136]	[22]		
**Conformity with other health care**	[33]		[33, 68, 166]	[38, 94]	[33, 68, 125, 166, 200]	[38, 51]	[33]		[201]
**Costs**	[110]	[22, 35, 49, 111-113]	[38, 54, 114-116]	[35, 44, 45, 87, 90, 99, 112, 115, 117-123]	[38, 46, 54, 70, 102, 114, 116, 122, 124-133]	[45, 50, 87, 104, 112, 117, 118, 121, 124, 127, 130, 132, 134-140]	[128]	[50, 134]	[108, 141-143]
**Culture**	[48]	[48, 145, 151, 218]		[64, 145, 151]		[145, 151, 152, 193]		[145, 151]	[143, 191]
**Education**	[70, 88, 95, 116, 178, 226]		[31, 46, 56, 88, 99, 154, 166, 226]	[56, 80]					[141]
**Holistic approach**	[74]	[74]	[211]	[211]		[150]			[66]

Category	Entity								
	P		HP		HS		Soc		All
	S	F	S	F	S	F	S	F	
**ICT training**	[88, 180, 202-204]	[31, 37, 49, 80, 181, 185, 186, 192, 202, 205, 206]	[22, 31, 53, 54, 84, 85, 88, 92, 149, 150, 207]	[36, 44, 80, 84, 85, 119, 122, 145, 163, 195, 207-209]	[22, 43, 150, 172]	[61, 63, 103, 151, 175, 193]	[210]	[175]	[71]
**ICT vs Traditional methods**	[74, 219-221]	[31, 74, 80, 192, 222]	[44, 46, 64, 68, 74, 130, 219, 220]	[38, 52, 74, 80, 93, 97, 117, 121, 130, 223]	[46, 57, 68, 130, 219, 220]	[38, 97, 121, 130, 223]	[219]		[201]
**Infrastructure**		[37, 111, 192]		[117, 155]	[43, 172]	[50, 138, 155, 193, 194]		[50, 138]	[152]
**Interoperability**	[22, 165]	[146]	[22, 40, 42, 44, 64, 68, 144, 166-168]	[42, 94, 95, 118, 121, 146, 155, 168, 169]	[22, 23, 40, 68, 102, 125, 126, 153, 154, 161, 166, 167, 170-172]	[51, 61, 62, 98, 121, 132, 136, 146, 150, 155, 173-175]	[22]		[66, 88, 106, 108, 112, 152, 164]
**Leadership**			[114, 122, 156, 168]	[168, 195]	[122, 140, 156, 158, 161, 196, 197]	[36, 104, 105, 137, 140, 198]			[69, 199]
**Legal**				[188]	[76, 230, 232]	[137, 138, 147, 163, 188, 230, 232]	[76]		[141]
**Patient empowerment and self-management**	[19, 22, 25, 27, 30, 33, 34, 47, 74, 75, 101, 111, 146, 176-182]	[28, 32, 35, 74, 75, 176, 179, 183-187]	[34, 52, 85, 86, 95, 114, 176]	[32, 35, 85, 87, 176, 188]	[59, 154, 189]	[32, 188]			[190, 191]
**Patient-provider relationship**	[30, 48, 49, 196]	[35, 48, 111, 218]	[30, 85, 99, 196]	[35, 80, 85, 87]	[196]				[69]
**Policies**	[110, 146]	[32, 135]	[38, 53, 56, 70, 119, 147, 148]	[32, 38, 119, 122, 148, 149]	[38, 43, 53, 102, 131, 147, 150-161]	[32, 38, 55, 132, 135, 146, 158, 159, 161-163]	[150, 151]		[32, 108, 128, 142, 164]

	Entity								
**Category**	**P**		**HP**		**HS**		**Soc**		**All**
	**S**	**F**	**S**	**F**	**S**	**F**	**S**	**F**	
**Privacy/security**	[27, 45, 116, 202, 203, 224, 225]	[27, 49, 111, 112, 186, 202, 203, 206, 213, 218, 220, 224-228]	[99, 116, 229]	[40, 64, 84, 85, 112, 117, 118, 148, 149, 209, 220, 226, 228]	[43, 116, 161, 230, 231]	[62, 63, 112, 173, 217, 220]	[203, 225]	[203, 225]	[109]
**Quality of healthcare**	[19, 28, 33, 34, 48, 49, 72, 77, 110, 116, 127, 182, 235, 236]	[22, 48, 49, 236]	[19, 25, 33, 34, 43, 52, 72, 76, 77, 84, 86, 91, 98, 99, 115, 116, 118, 119, 127, 148, 166, 195, 229, 235]	[22, 56, 84, 88, 115, 163]	[33, 34, 72, 76, 77, 96, 102, 116, 127, 181, 216, 234, 235]	[22, 63]	[33, 76, 77, 235]		[29, 66, 107, 141]
**Reliability connection/technology**	[212, 213]	[22, 37, 192]	[42, 214]	[22, 42, 64, 112, 123]	[214]	[22, 150, 215]		[22]	[65, 67]
**Safety**	[53, 165, 219]	[111, 220]	[53, 219, 233]	[118, 220]	[90, 219]	[220]	[219]		[29]
**Standardisation**	[45]		[76, 89]	[95, 149]	[76, 126, 132, 150, 152, 174, 216]	[39, 50, 95, 149, 173, 174, 216, 217]	[76, 150]		
**System architecture**	[20, 25, 26, 29, 47]	[19, 20, 22, 28, 32, 47]	[25, 29, 85]	[19, 22, 32, 85]	[92, 144]	[22, 32, 103, 134, 145]			
**Usability**	[19-31]	[20, 32-37]	[19, 22, 25, 29-31, 38-44]	[32, 34, 35, 45]	[22, 38-41, 46]	[32, 34]	[22]		
**Workflow**	[28, 36, 67, 72-79]	[19, 32, 75, 78, 80-83]	[25, 36, 39, 64, 67, 72, 73, 76, 77, 84-92]	[19, 22, 32, 38, 56, 80, 84, 85, 88, 92-99]	[39, 67, 73, 76, 77, 88, 90, 92, 100-102]	[22, 32, 38, 88, 97, 98, 103-105]	[73, 77, 100]		[69, 71, 106-109]
