# Supplementary material for: Factors Determining the Success and Failure of eHealth Interventions: Systematic Review of the Literature
Source: J Med Internet Res. 2018 May 1;20(5):e10235. doi: 10.2196/10235 (PMC5954232; doi:10.2196/10235)
Supplement: Multimedia Appendix 2 [file jmir_v20i5e10235_app2.pdf]

Article frequency (AF)and unique article frequency (UAF) per category, article frequency as success (AFS) and failure (AFF) per category, and article frequency as success (S) and failure (F) per entity [Patient (P), Health care professionals (HP), Health system (HS), and Society (Soc)] per category.

					P		HP		HS		Soc	
**Category**	**AF**	**UAF**	**AFS**	**AFF**	**S**	**F**	**S**	**F**	**S**	**F**	**S**	**F**
**Access to healthcare**	33	25	20	11	9	2	9	5	2	3	0	1
**Adherence to treatment**	6	6	4	1	3	1	1	0	0	0	0	0
**Adoption**	39	31	15	17	4	4	8	7	2	6	1	0
**Assessment**	6	2	4	2	1	0	1	1	1	1	1	0
**Conformity with other health care**	15	9	10	4	1	0	3	2	5	2	1	0
**Costs**	71	50	25	**42**	1	6	5	15	**18**	**19**	1	**2**
**Culture**	16	9	1	13	1	4	0	3	0	4	0	**2**
**Education**	17	14	14	2	6	0	8	2	0	0	0	0
**Holistic approach**	6	4	2	3	1	1	1	1	0	1	0	0
**ICT training**	53	43	21	31	5	11	11	13	4	6	1	1
**ICT vs Traditional methods**	40	22	19	20	4	5	8	10	6	5	1	0
**Infrastructure**	15	12	2	12	0	3	0	2	2	5	0	2
**Interoperability**	58	45	28	23	2	1	10	9	15	13	1	0
**Leadership**	21	17	11	8	0	0	4	2	7	6	0	0
**Legal**	13	9	4	8	0	0	0	1	3	7	1	0
**Patient empowerment and self-management**	52	40	30	20	**20**	12	7	6	3	2	0	0
**Patient-provider relationship**	18	12	9	8	4	4	4	4	1	0	0	0
**Policies**	53	36	29	19	2	2	7	6	**18**	11	2	0
**Privacy/security**	55	38	17	37	7	**16**	3	13	5	6	2	**2**
**Quality of healthcare**	71	44	**55**	12	14	4	**24**	6	13	2	**4**	0
**Reliability connection / technology**	19	14	5	12	2	3	2	5	1	3	0	1
**Safety**	15	9	9	5	3	2	3	2	2	1	1	0
**Standardisation**	22	15	12	10	1	0	2	2	7	8	2	0
**System architecture**	25	15	10	15	5	6	3	4	2	5	0	0
**Usability**	46	28	33	13	13	7	13	4	6	2	1	0
**User involvement**	35	22	20	10	5	2	8	5	6	3	1	0
**Workflow**	**83**	**51**	43	34	11	8	18	**17**	11	9	3	0
